# Overexpression of TaPIP1A enhances drought and salt stress tolerance in Arabidopsis: cross-species conservation and molecular dynamics

**DOI:** 10.3389/fpls.2024.1425700

**Published:** 2025-06-02

**Authors:** Jie Han, Wei Zhang, Liya Zhi, Silong Chen, Hui Zhao, Xinyu Zhang, Liuge Xue, Jun Ji, Fengzhi Wang, Junming Li

**Affiliations:** ^1^ Hebei University of Science and Technology, Shijiazhuang, China; ^2^ Hebei University of Economics and Business, Shijiazhuang, China; ^3^ Center for Agricultural Resources Research, Institute of Genetics and Developmental Biology, Chinese Academy of Sciences, Shijiazhuang, China; ^4^ Ministry of Education Key Laboratory of Molecular and Cellular Biology, Hebei Collaboration Innovation Center for Cell Signaling, Hebei Key Laboratory of Molecular and Cellular Biology, College of Life Sciences, Hebei Normal University, Shijiazhuang, China; ^5^ Hebei Key Laboratory of Crop Salt-Alkali Stress Tolerance Evaluation and Genetic Improvement, Cangzhou Academy of Agriculture and Forestry Sciences, Cangzhou, China

**Keywords:** wheat, *TaPIP1A*, abiotic stress, transcriptional regulation, protein-protein interactions, *TaWRKY71*, crop resilience, plant stress responses

## Abstract

Understanding the mechanisms underlying plant responses to abiotic stress is crucial for improving crop resilience. This study characterizes the role of *TaPIP1A*, a member of the plasma membrane intrinsic proteins (PIPs) in wheat, highlighting its critical function in mediating stress responses. We demonstrate that *TaPIP1A* enhances tolerance to salt and drought stress through specific protein-protein interactions and transcriptional regulation. In *TaPIP1A* knockout wheat strains, there was a significant decrease in stress tolerance, whereas Arabidopsis plants overexpressing *TaPIP1A* exhibited marked improvements in survival under similar stress conditions. We also discovered that *TaPIP1A* interacts with TaPIP2-3 and is regulated by the transcription factor *TaWRKY71*, which binds to its promoter to activate gene expression. These findings contribute to our understanding of the molecular mechanisms by which PIPs facilitate plant adaptation to environmental stresses, offering new avenues for the development of stress-resistant crop varieties. Our research provides insights into the genetic manipulation of intrinsic membrane proteins to enhance plant stress tolerance, a step forward in agricultural biotechnology.

## Introduction

Non-biological stresses, particularly high salinity and drought, have significantly impacted the growth and productivity of most major crops (Chandrasekaran et al., 2021; Alavilli et al., 2023). With global climate change and deteriorating environmental conditions, these stressors pose increasingly severe challenges to agricultural production (Halder et al., 2022; Li et al., 2022a; Kholssi et al., 2023). Therefore, the identification and utilization of novel genes that enhance crop tolerance to non-biological stresses are crucial for crop breeding and ensuring food security (Zhang et al., 2020; Thudi et al., 2021; Zhang et al., 2022). In this context, the intrinsic membrane protein (PIP), which serves as a channel protein facilitating water passage through biological membranes, has attracted widespread attention in the research community (McGaughey et al., 2021; Scochera et al., 2022; Al-Mualem et al., 2023).

Based on evolutionary analysis, PIP can be classified into two subtypes, PIP1 and PIP2, with PIP1 being more strictly conserved due to a longer N-terminus, shorter C-terminus, and shorter extracellular loop A, exhibiting sequence similarities exceeding 90% (Li et al., 2019). While all plant PIP2 exhibit significant water permeability, PIP1 often shows lower activity or remains inactive, yet actively participates in controlling many crucial physiological traits in plants, such as drought and salt tolerance, cold resistance, growth and development, fruit ripening, and seed germination (De Vega et al., 2021; Berna-Sicilia et al., 2023). Moreover, the protein interaction between PIP1 and PIP2 leads to the relocation of PIP1 from the endoplasmic reticulum membrane to the plasma membrane, regulating its water permeability. Therefore, identifying PIP2 interacting with PIP1 and exploring their interaction mechanisms are essential for understanding how plants modulate their responses to non-biological stresses (Patankar et al., 2019; De Vega et al., 2021; Berna-Sicilia et al., 2023).

In regulating plant responses to non-biological stresses, transcription factors play a central role (Zhao et al., 2022; Baoxiang et al., 2023). Particularly, the WRKY transcription factor family regulates plant responses to non-biological stresses by binding to W-box cis-acting elements in the target gene promoters (Abdullah-Zawawi et al., 2021; Wani et al., 2021; Wang et al., 2023a). Previous studies have shown that overexpression of *TaWRKY71* enhances tolerance to high salt and drought-induced stresses in Arabidopsis, highlighting the potential importance of WRKY transcription factors in plant stress responses. However, further clarification is needed regarding the transcriptional regulatory mechanisms of these genes, especially their roles in wheat.

Hence, in this study, three PIP1 genes were cloned from wheat and characterized through gene sequencing, chromosome mapping, and analysis of their expression patterns. We employed physiological, molecular, and genetic approaches to elucidate the association between *TaPIP1A* and wheat plants’ tolerance to high salt and drought-induced stresses (Zhang et al., 2023). Additionally, PIP2 interacting with *TaPIP1A* was identified, and the transcriptional regulatory mechanisms of their genes were investigated to reveal the molecular network regulating wheat’s response to high salt and drought stresses, providing potential genetic resources and molecular markers to enhance crop stress tolerance.

The results of this study demonstrate the central role of the *TaPIP1A* gene in wheat’s response to high salt and drought stresses and the importance of its interaction with TaPIP2-3 in maintaining cell water balance and membrane integrity. By deeply analyzing the regulatory mechanism of *TaWRKY71* transcription factor on *TaPIP1A* gene expression, we not only enhance the understanding of plant regulation networks in response to non-biological stresses but also lay the foundation for further exploration of similar regulatory mechanisms in other crops. This study emphasizes the importance of precisely regulating gene expression through genetic engineering in crop improvement programs, providing theoretical and practical foundations for developing new varieties with high salt and drought tolerance.

Moreover, by uncovering the complex interaction network between transcription factors and water channel proteins, this study opens new directions for plant biotechnology research. These findings are not only significant in the field of agricultural science but also offer a new perspective for basic plant biology research, particularly in understanding how plants adapt to environmental stresses by finely regulating intracellular water transport and membrane stability.

In conclusion, this study not only provides new ideas and methods to enhance the stress tolerance of important crops like wheat but also offers valuable insights into the molecular mechanisms of plant responses to non-biological stresses. As the impact of global climate change and non-biological stresses on agricultural production becomes increasingly significant, conducting such research holds profound implications for promoting sustainable agricultural development and ensuring global food security.

## Materials and methods

### Experimentation with plant material and stress treatment

In this study, we used EMS (ethyl methanesulfonate) mutagenesis method to obtain the experimental wheat seeds from the Kenong9204 mutant library. This mutant library was jointly established by the Institute of Genetics and Developmental Biology of the Chinese Academy of Sciences and Hebei Normal University, covering a wide range of gene mutations (Wang et al., 2023b). Initially, a gene knockout mutant library of KN9204 was constructed following the description by Zhang et al. (2017) (Zhang et al., 2017), from which the pip1a mutant was screened. By employing specific primers mPIP1AF/R (**Supplementary Table 1**) for PCR screening of leaf and root samples from 4332 M3 generation seedlings, a total of three independent pip1a mutant lineages were identified and subsequently backcrossed twice with the wild-type KN9204.

To evaluate the expression patterns of stress-responsive genes in these plants, KN9204 plants were hydroponically cultivated at 25°C under a 16-hour light/8-hour dark photoperiod until heading. At 10 days post-germination, the plants were subjected to different stress treatments including 100 mM NaCl, 20% PEG-6000, 10 mM H_2_O_2_, and 100 mM ABA. Leaf and root samples were collected at 0, 1, 3, 6, 12, and 24 hours post-treatment with each stress repeated thrice.

Furthermore, to analyze stress resistance in transgenic Arabidopsis plants expressing *TaPIP1A*, seeds were germinated on basic MS medium or MS medium supplemented with 200 mM NaCl or 300 mM mannitol to induce stress. Germination was recorded daily for nine days starting from seed imbibition. For seedling stress resistance assessment, 3-day-old seedlings were transferred to solid MS medium with 0 or 100 mM NaCl and grown for 8 days. For adult plant stress resistance assessment, 4-week-old plants were transferred to pots containing vermiculite and soil (2:1 ratio) or soil supplemented with 300 mM NaCl, followed by 8 days of cultivation, after which plant photographs were taken, and all leaves were collected, with each treatment replicated thrice (Tian et al., 2013).

Moreover, stress treatments were applied to KN9204 and its *TaPIP1A* gene knockout mutants. Using a hydroponic culture system, 10-day-old wild-type and mutant seedlings were transferred to half-strength Hoagland solution supplemented with NaCl or PEG to achieve final concentrations of 200 mM or 30%, and were cultivated at 22°C for 8 days before imaging. For soil-based experiments, 10-day-old wild-type and mutant seedlings were transplanted to pots containing vermiculite and soil (2:1 ratio) and subjected to high-stress inducing concentrations of 400 mM NaCl or 40% PEG, then grown for 15 days before imaging (Dobrikova et al., 2017). All experiments were repeated three times to ensure result reliability.

### Chromosomal localization and functional domain analysis of the Pip1 gene family in the wheat genome

Using wheat genome sequence data sourced from the Ensembl Genomes database (https://ftp.ensemblgenomes.ebi.ac.uk/) and the sequence alignment tool BLAST (https://blast.ncbi.nlm.nih.gov/), this study successfully identified homologous sequences of the TaPIP1 gene family members. In order to verify the exact location of these genes within the wheat genome, multiple BLAST searches were conducted, along with several rounds of independent sequence alignments for the suspected gene sequences, ensuring the accuracy and reliability of the positional information obtained.

By submitting the protein sequences encoded by the TaPIP1 genes to the InterPro database (https://www.ebi.ac.uk/interpro/), this research identified the conserved functional domains and structural features of the TaPIP1-encoded proteins. InterPro is an integrated protein function annotation database that collates information on protein families, structural domains, and functional sites from various databases and resources, offering biologists and bioinformaticians a comprehensive platform for protein function prediction and annotation. The principle is to analyze protein sequences based on different protein identifiers, such as UniProtKB, and map them to known protein families, structural domains, and functional modules. This study provided Gene Ontology (GO) annotations for the TaPIP1 family-encoded proteins through InterPro to reveal their functions in biological processes such as stress response and drought tolerance.

### Cloning and sequence analysis of the TaPIP1 gene in wheat

This study provides a detailed methodological investigation into the cloning and sequence analysis of the TaPIP1 gene in the wheat variety KN9204. Initially, total RNA was extracted from KN9204 leaves using TRIzol^®^ reagent (Life Technologies, California, USA), followed by first-strand cDNA synthesis with AMV reverse transcriptase (TaKaRa, Dalian, China). Simultaneously, genomic DNA was extracted from KN9204 using a plant genome DNA extraction kit (TIANGEN, Beijing, China), with all procedures strictly following the manufacturers’ protocols. Through PCR directional cloning based on expressed sequence tags (ESTs), utilizing primers TaPIP1F and TaPIP1R (**Supplementary Table 1**), the full-length gDNA and cDNA of the TaPIP1 gene were successfully obtained. Subsequently, we quantified the obtained DNA using a Nanodrop spectrophotometer (Thermo Scientific, USA) and assessed the purity and size of the nucleic acids through 1% agarose gel electrophoresis (Harrington, 1993). Furthermore, the promoter regions of homologous TaPIP1 genes were cloned using different primers: *TaPIP1A*-promoterF/R for *TaPIP1A*, *TaPIP1B*-promoterF/R for *TaPIP1B*, and *TaPIP1D*-promoterF/R for *TaPIP1D* (**Supplementary Table 1**). Subsequently, the corresponding amino acid sequences were deduced using Lasergene software (DNASTAR, Madison, Wisconsin, USA), and conserved domains were identified with the SMART motif search tool (http://smart.embl.de/). A phylogenetic tree was constructed using the MEGA 6.0.6 program based on the Neighbor-Joining method. Through the aforementioned techniques, we not only successfully cloned members of the TaPIP1 gene family and analyzed their sequence characteristics but also further elucidated the potential functions and evolutionary relationships of these genes in wheat. This research methodology provides crucial technical support for a deeper understanding of the role of the TaPIP1 gene in plant stress response in wheat.

### Expression of *TaPIP1A* gene and quantitative analysis of stress-responsive genes in transgenic Arabidopsis plants

To investigate the expression pattern of the *TaPIP1A* gene in wheat and its impact on stress response in transgenic Arabidopsis plants, this study evaluated the expression levels of *TaPIP1A* and 16 stress-responsive genes using quantitative real-time polymerase chain reaction (qRT-PCR) technology. Specific primers listed in **Supplementary Table 2** were employed for quantification of *TaPIP1A* gene in wheat and stress-responsive genes in transgenic Arabidopsis plants, with TaActin (AB181991) and AtActin (*At3g18780*) genes used as internal controls. The qRT-PCR experiments were conducted on the ABI PRISM^®^ 7500 RT-PCR system (Applied Biosystems™, Waltham, Massachusetts, USA) using SYBR Premix Ex Taq™ II (TaKaRa, Dalian, China) following the manufacturer’s instructions. Quantitative comparison analysis of mRNA transcript levels was performed using the 2^−ΔΔCT^ method. This approach not only accurately assessed the expression of the *TaPIP1A* gene in wheat but also revealed its role in stress response in transgenic Arabidopsis plants, providing important molecular evidence for understanding the function of the *TaPIP1A* gene in plant stress responses. The application of this method deepened our understanding of the mechanisms by which the *TaPIP1A* gene acts in stress response, laying a theoretical foundation for future genetic enhancements to improve crop stress tolerance.

### Construction of vector and transformation in *Arabidopsis thaliana*

To analyze gene function, this study initially amplified the coding sequence (CDS) of *TaPIP1A* using gene-specific primers XbaI-PIP1AF and BamHI-PIP1AR and cloned it into the pCAMBIA 1300 vector for expression under the control of CaMV 35S promoter. Subsequently, the vector containing 35S::*TaPIP1A* construct was transformed into Arabidopsis plants using the floral dip method as described by Clough and Bent (Clough and Bent, 1998). Transgenic plants were obtained through selection on half-strength solid MS medium containing 25 mg/mL hygromycin (Krogan et al., 2012). Further analysis was carried out using T3 homozygous plants. To investigate the cellular localization of *TaPIP1A* protein, its CDS was inserted into the pEGAD-GFP vector and expressed under the control of the CaMV 35S promoter. The 35S::GFP vector was used as a control simultaneously.

### The localization of *TaPIP1A* protein in tobacco and Arabidopsis cells

To investigate the precise subcellular localization of the *TaPIP1A* protein in plant cells, tobacco (*Nicotiana benthamiana*) and Arabidopsis leaves were chosen as transformation materials in this experiment. The transformation was carried out using the *Agrobacterium tumefaciens* strain GV3101-mediated method, where the pEGAD empty vector and the pEGAD-*TaPIP1A* vector containing *TaPIP1A* were separately introduced into the mentioned plants. Following transformation, fluorescence signals in the leaf tissues of tobacco and the epidermal cells of Arabidopsis roots were measured using the SP8 confocal microscope manufactured by Leica Microsystems. This step aimed to indirectly determine the distribution of *TaPIP1A* protein within the cells by observing the localization of the green fluorescent protein (GFP).

### Determination of plant physiological parameters

To comprehensively assess plant physiological indicators, various methods were employed in this study to determine the ionic leakage (IL) of cell membranes, chlorophyll content, relative water content (RWC), proline content, and malondialdehyde (MDA) content. Initially, leaf samples were cut into 0.5 mm strips, incubated overnight in 10 ml of ultrapure water at room temperature, then rinsed three times with ultrapure water and the initial electrical conductivity (C1) was measured using the Orion Lab Star EC112 conductivity probe (Thermo Fisher). Subsequently, the samples were boiled for 30 minutes, cooled to room temperature, and the final electrical conductivity of the solution (C2) was measured. The ionic leakage was evaluated using the formula IL(%) = (C1/C2) × 100%. The determination of chlorophyll content followed Arnon’s previous description. Briefly, chlorophyll content was measured by quantifying the absorption of chlorophyll acetone extracts in 80% aqueous solution using a spectrophotometer. The concentrations of chlorophyll a and b were measured in 10 mm cuvettes at 663 and 645 nm wavelengths. The determination of RWC involved weighing leaf samples immediately to obtain fresh weight (FW), soaking them in distilled water for 4 hours to obtain turgid weight (TW), and then drying them in an oven at 80°C for 24 hours to obtain dry weight (DW). RWC was calculated using the formula RWC (%) = [(FW - DW)/(TW - DW)] × 100% (Sade et al., 2015). The proline and MDA content were assessed using kits from the Nanjing Jiancheng Bioengineering Institute following the manufacturer’s instructions. These comprehensive analytical methods not only accurately assess the physiological changes in plants but also provide important biochemical indicators for the plant’s growth status and responses to stressors.

### Protein immunoblot analysis

In this study, total proteins were extracted from approximately 100 mg of *Nicotiana benthamiana* tobacco leaves using a lysis buffer containing 50 mM Tris pH 7.5, 150 mM NaCl, 0.1% Triton X-100, 100 μg/mL PMSF, and Roche protease inhibitor cocktail (He et al., 2014). Next, the homogenate was centrifuged at 13,500 g to collect the supernatant, which was then heated and boiled for 5 minutes in 2× SDS protein loading buffer. Protein separation from the homogenate was carried out using 12.5% SDS-PAGE, and the proteins were transferred to a PVDF membrane using Bio-Rad’s Mini-Protean II system via semi-dry electroblotting technique. Verification of successful protein transfer and sample uniformity was performed by Ponceau S staining of the membrane. Subsequently, detection of the GFP-fusion protein was conducted using the HRP-conjugated anti-GFP ab6663 antibody from Abcam (diluted at 1:2000) (Ren et al., 2021). A molecular weight standard, Thermo Scientific’s 170 kD PagerulerTM protein marker, was utilized for size determination.

### Isolation of protoplasts from wheat seedlings for BiFC analysis

In this study, protoplasts were isolated from 10-day-old wheat seedling leaves grown in darkness, following a method similar to that used for *Arabidopsis thaliana* (He et al., 2006). Gene-specific primers were used to amplify the CDS of *TaPIP1A* and TaPIP2-3. *TaPIP1A* was amplified using BamHI-PIP1AF and StuI-PIP1AR primers, while TaPIP2-3 was amplified using BamHI-PIP2F and StuI-PIP2R primers. Subsequently, these sequences were cloned into pxcGFP, pcYFP, and pnYFP vectors, generating the constructs *TaPIP1A*::cGFP, TaPIP2::cGFP, BiFC-*TaPIP1A*::cYFP, and BiFC-TaPIP2::nYFP. These constructs were then transformed into wheat leaf mesophyll protoplasts for bimolecular fluorescence complementation (BiFC) analysis. Fluorescence was observed using a Leica Microsystems SP8 confocal microscope. The process encompassed protoplast preparation, gene cloning, construct expression, transformation, and detailed steps for fluorescence observation, ensuring the experiment’s efficiency and reproducibility.

### Investigating the interaction between *TaPIP1A* and TaPIP2-3 using the yeast two-hybrid system

To explore the interaction between *TaPIP1A* and TaPIP2-3, the current study employed the MatchmakerTM GAL4 yeast two-hybrid system (Clontech Laboratories, Mountain View, California, USA) following the provided protocol. Initially, the CDS of *TaPIP1A* were amplified using the EcoRI-PIP1AF and BamHI-PIP1AR primers, and simultaneously, the CDS of TaPIP2-3 were amplified using the EcoRI-PIP2F and BamHI-PIP2R primers. Subsequently, these CDS were individually cloned into the pGADT7 and pGBKT7 vectors. For the positive control experiments, pGADT7-T and pGBKT7-53 vectors were co-transformed, while for the negative controls, pGADT7-T and pGBKT7-Lam vectors were co-transformed. This approach effectively facilitated the identification of whether a direct interaction exists between these two proteins, thereby offering crucial insights into their functionality in wheat growth and development.

### Analysis of EMSA

To investigate the DNA binding activity of *TaWRKY71* protein, the CDS of *TaWRKY71* was first amplified using BglII-WRKY71F and SalI-WRKY71R primers, cloned into the pMALC2X vector, generating the MBP-*TaWRKY71* fusion construct. These constructs were subsequently transformed into BL21 competent cells. The MBP and MBP-*TaWRKY71* fusion proteins were purified using amylose resin provided by New England Biolabs following a standardized protocol.

Subsequently, EMSA experiments were conducted using the LightShift™ Chemiluminescent EMSA Kit from Thermo Scientific with biotin-labeled probes. The binding activity of the fusion proteins was analyzed using oligonucleotide probes containing the TGAC motif from the *TaPIP1A* promoter, biotinylated at the 5’ end (Life Technologies Corporation, USA). The sequences of the synthesized oligonucleotide probes and their complementary probes are mentioned in **Supplementary Table 1**. The double-stranded DNA was synthesized by heating at 95°C for 5 minutes, followed by annealing at 4°C in annealing buffer containing 10 mM Tris-HCl pH 7.6, 1 mM EDTA, 50 mM NaCl.

The protein-probe mixtures were separated on a 6% SDS-PAGE gel, transferred onto a nylon membrane (GE Healthcare, UK), and then incubated at room temperature for 25 minutes. The migration of biotin-labeled probes was determined using the Chemiluminescent Nucleic Acid Detection Module from Thermo Scientific following the manufacturer’s recommended protocol.

### Evaluation of *TaPIP1A* transcript activation ability in tobacco leaves

To assess the transcription activation ability of *TaPIP1A* in tobacco leaves, this study first amplified a 2 kb-long *TaPIP1A* promoter using specific primers PstI-PIP1ApromoterF and NcoI-PIP1ApromoterR (containing PstI and NcoI restriction enzyme sites, **Supplementary Table 1**) via PCR. The amplified fragment was then cloned into the expression vector pCAMBIA 1391 to generate the promoter PIP1AF::GUS reporter gene construct. Subsequently, the CDS of *TaWRKY71* was amplified from cDNA using specific primers BglII-WRKY71F and XbaI-WRKY71R (**Supplementary Table 1**) and cloned into the pEGAD vector. The promoter reporter gene construct (PIP1AF::GUS reporter) and effector constructs (pEGAD and pEGAD-WRKY71) were then transformed into tobacco leaves using *Agrobacterium tumefaciens* strain GV3101 via infiltration.

GUS enzyme activity was determined using a dual monitoring method of histochemical and fluorescence assays. For histochemical GUS staining, the method described by Van der Does et al. was followed (Van der Does et al., 2013). In the histochemical GUS experiments, each construct was tested in at least ten different transformations. The protein content in the supernatant was quantified using the Bradford method (Bradford, 1976). The fluorescence method for measuring GUS activity followed the protocol outlined by Bihmidine et al (Bihmidine et al., 2013), where GUS activity was expressed as nanomoles of 4-methylumbelliferone (MU) produced per minute per milligram of protein. Each measurement was repeated three times to obtain the average GUS activity of five independent transformations.

### Statistical methods

This study utilized SPSS 19.0 software (Chicago, USA) for statistical analysis to evaluate the significance differences in the experimental data. An independent samples t-test was employed to determine the significance level when the test samples were exposed to the same salt concentrations, with double asterisks (**) and single asterisk (*) denoting significance at the *P* = 0.01 and *P* = 0.05 levels, respectively. For comparisons between different treatments, a two-way analysis of variance (ANOVA) followed by Tukey’s multiple comparison test was conducted, where uppercase letters denoted significant differences among all treatments at the *P* = 0.01 level. Each experiment was replicated at least three times, and the average standard deviation for the three repetitions was indicated by vertical bars. This method facilitated an accurate assessment of the impact of experimental treatments on the samples and provided statistical confirmation of the reliability of the results.

## Results

### Discovery of the wheat *TaPIP1A* gene encoding PIP1 subfamily protein in bioinformatics

**Figure 1:**
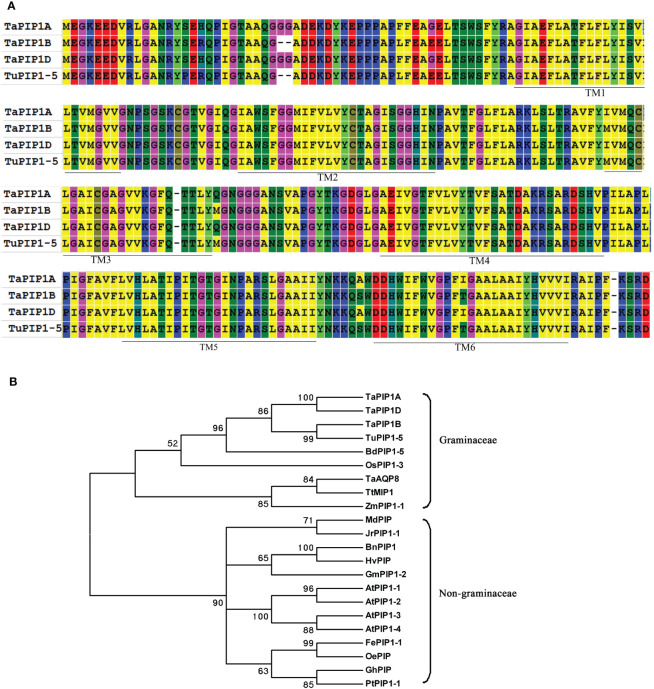
Phylogenetic Analysis of PIP1 Subfamily Aquaporin Proteins. **(A)** Sequence alignment of PIP proteins from hexaploid wheat and T. urartu. The amino acids highlighted in the same color indicate identical residues, while those in different colors represent variations. The six putative transmembrane regions (TM1–6) in PIP1 are marked with solid lines above the sequence; **(B)** Phylogenetic tree of TaPIP1 and its homologous proteins from different species. The full-length amino acid sequences of PIP1 were aligned using MEGA 6.06 software, and the phylogenetic tree was constructed using the neighbor-joining method with 1,000 bootstrap replicates.

Through genome mapping analysis, we found that the Pip1 gene family exhibits different distribution densities on different chromosomes of wheat, with the majority of Pip1 genes concentrated on chromosomes 2, 5, and 6 (**Supplementary Figure 1A**). Using the sequence of the homologous gene in rice (*Os02g0823100*), the TaPIP1 gene was cloned and identified as contig_5785149 in the wheat URGI BLAST database. Three homologous genes belonging to the A, B, and D subgenomes were cloned from the wild-type wheat KN9204 using TaPIP1F/R primers, with genomic sequences of 1124 bp, 1076 bp, and 1125 bp, and CDS of 879 bp, 873 bp, and 879 bp, respectively, named *TaPIP1A*, *1B*, and *1D* (accession numbers KC700309, KU366812, and KU366814). Similar to most species, the TaPIP1 gene comprises three exons and two introns. Amino acid sequence alignment revealed homologies of 97% and 100% between *TaPIP1A* and *TaPIP1B*/D, all possessing six transmembrane domains (**Figure 1A**). Notably, there were only 23 single nucleotide polymorphisms (SNPs) between *TaPIP1A* and *TaPIP1D*. Additionally, *TaPIP1B* showed significant sequence divergence from *TaPIP1A* and *TaPIP1D*, but exhibited 100% homology with *TuPIP1-5* from the Triticeae ancestor species *Triticum urartu* (EMS53768). Through functional domain analysis, it was revealed that the protein encoded by *TaPIP1A* belongs to the major intrinsic protein (MIP) superfamily and aquaporin (aqp) family, with a typical aquaporin protein structure (https://www.ebi.ac.uk/interpro/structure/PDB/3gd8/). Its functional annotation indicates that it plays a role in the regulatory pathway of plants responding to long-term water deficiency and is closely related to drought tolerance. Specifically, its GO terms include “response to water deprivation” (GO:0009414) in Biological Process and “water channel activity” (GO:0015250) in Molecular Function (**Supplementary Figure 1B**). These annotations suggest the important role of *TaPIP1A* in the response and regulation of plants to changes in water availability, potentially serving as a key component of drought tolerance mechanisms.

A phylogenetic tree of 22 PIP1 homologous proteins from different species was constructed (**Figure 1B**). The highest similarity was observed between the three TaPIP1 proteins and TuPIP1-5, followed by BdPIP1-5 and OsPIP1-3. Evolutionarily, TaPIP1 is closely related to the PIP1 subfamily, indicative of substantial sequence homology among different species. Furthermore, TaPIP1 shares higher homology with Poaceae plant proteins compared to non-Poaceae species. For instance, *TaPIP1A* exhibited 90.8% identity with OsPIP1-3 in rice but only about 74.6% identity with AtPIP1B in Arabidopsis. Through bioinformatics analysis, we elucidated the homology and evolutionary relationships of the *TaPIP1A* gene and its encoded PIP1 subfamily protein in wheat, leading to a successful cloning.

### Stress-induced expression of *TaPIP1A* in plasma membrane and nuclear membrane demonstrated *in vitro* experiments

**Figure 2:**
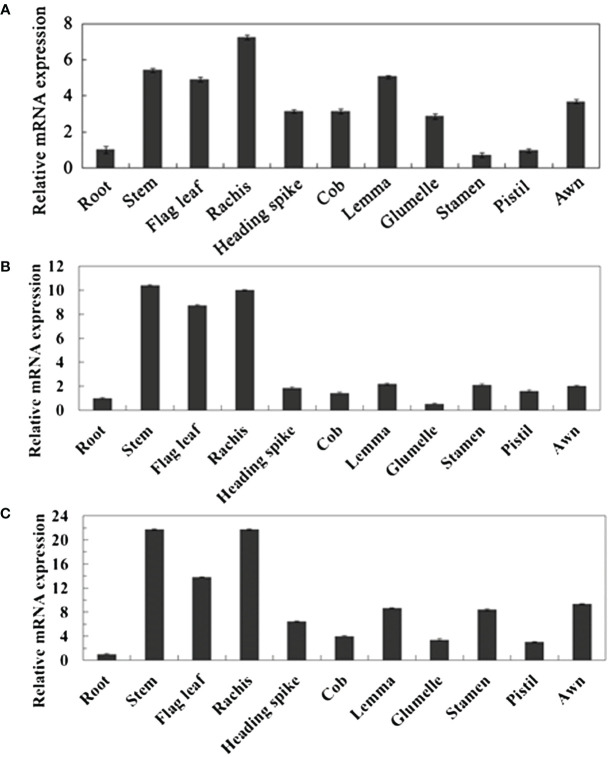
Tissue-Specific Expression Analysis of TaPIP1 Genes in Wheat at Heading Stage. **(A)** *TaPIP1A*; **(B)** *TaPIP1B*; **(C)** *TaPIP1D*. The reference gene used was wheat TaActin gene. All other values were measured relative to the expression of TaActin in roots. Vertical bars represent standard deviations (SD). Each experiment was repeated three times.

**Figure 3:**
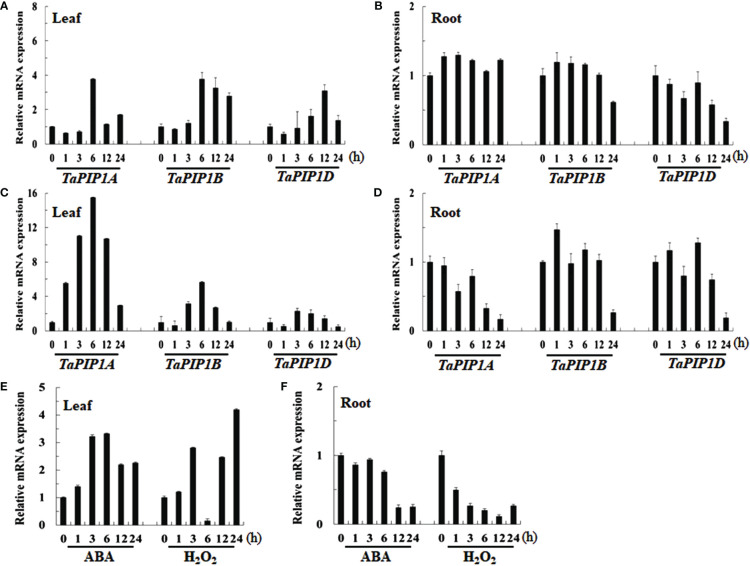
Expression Analysis of *TaPIP1A* in Wheat under Different Stress Induction Treatments Using qRT-PCR. The plants were cultivated under normal moisture conditions as a control. Analysis of *TaPIP1A*, 1B, and 1D expression in the leaves **(A)** and roots **(B)** of 10-day-old wheat seedlings under salt stress; as well as in the leaves **(C)** and roots **(D)** under PEG-induced stress. Analysis of *TaPIP1A* expression in the leaves **(E)** and roots **(F)** of 10-day-old wheat seedlings under ABA or H_2_O_2_-induced stress. The vertical axis indicates fold change, while the horizontal axis indicates treatment time (0, 1, 3, 6, 12, and 24 hours). TaActin in wheat was used as an internal control. Vertical bars represent standard deviation (SD). Each experiment was conducted in triplicate, and all replicates yielded similar results. Note: Plants were grown under normal moisture conditions as the control. Expression analysis of *TaPIP1A*, *1B*, and *1D* in the leaves **(A)** and roots **(B)** of 10-day-old wheat seedlings under salt stress was conducted, as well as in leaves **(C)** and roots **(D)** under PEG-induced stress. The expression analysis of *TaPIP1A* in the leaves **(E)** and roots **(F)** of 10-day-old wheat seedlings under ABA or H_2_O_2_-induced stress was also performed. The vertical axis indicates fold change, while the horizontal axis represents treatment time (0, 1, 3, 6, 12, and 24 hours). TaActin of wheat was used as an internal control. Vertical bars represent standard deviation (SD). Each experiment was triplicated, and all replicates yielded consistent results.

By collecting and analyzing various organ tissues of wheat plants at the heading stage, we investigated the expression patterns of *TaPIP1A*, 1B, and 1D in different wheat organs. The qRT-PCR analysis revealed that the expression levels of these three genes in aboveground parts such as stems, flag leaves, and spikes were significantly higher than in the roots (**Figure 2**). To examine the response of these genes to stress induction, 10-day-old wheat seedlings were subjected to osmotic stress (100 mM NaCl), water deficit stress (20% PEG-6000), 10 mM H_2_O_2_, and 100 μM ABA treatments. Furthermore, the plants were cultivated under normal moisture conditions as a control group. The expression of these three genes was induced by osmotic and water deficit stresses in leaves, but not in roots (**Figures 3A–D**). Transcripts of *TaPIP1A* and *1B* peaked at 6 hours post osmotic stress, while the peak for *TaPIP1D* occurred at 12 hours (**Figure 3A**). Under water deficit stress, *TaPIP1A* expression was significantly induced early at 1 hour (5.5-fold) and reached 15.5-fold induction at 6 hours. The induction of *TaPIP1B* and *1D* under PEG-6000 stress was relatively milder compared to *TaPIP1A* (**Figure 3C**). Therefore, subsequent studies focused mainly on *TaPIP1A*. ABA treatment also induced the expression of *TaPIP1A* in leaves, peaking at 3 hours (3.2-fold) (**Figure 3E**). Under H_2_O_2_ treatment, *TaPIP1A* expression in leaves peaked at 3 hours (2.8-fold), rapidly decreased, and a second peak was detected after 24 hours of continuous treatment (4.2-fold) (**Figure 3F**). These results indicate that the expression of *TaPIP1A* can be induced by NaCl, PEG, ABA, and H_2_O_2_ treatments.

To determine the subcellular localization of *TaPIP1A*, *TaPIP1A* was fused with GFP and expressed in the leaves of tobacco (*N. benthamiana*) and Arabidopsis plants. The transient expression of *TaPIP1A* in tobacco leaf mesophyll cells and stable expression in Arabidopsis revealed that *TaPIP1A* is specifically localized to the plasma membrane (PM) and nuclear membrane (NM), whereas the control GFP was distributed throughout the cell (**Supplementary Figure 2**). We confirmed the significant role of the *TaPIP1A* gene in salt and cold tolerance processes in wheat, showing its expression is regulated by environmental stress, and the specific localization of *TaPIP1A* protein in the plasma and nuclear membranes provides crucial insights for further exploration of its role in the wheat stress response mechanism.

### Deletion of *TaPIP1A* increases salt and drought sensitivity in wheat

**Figure 4:**
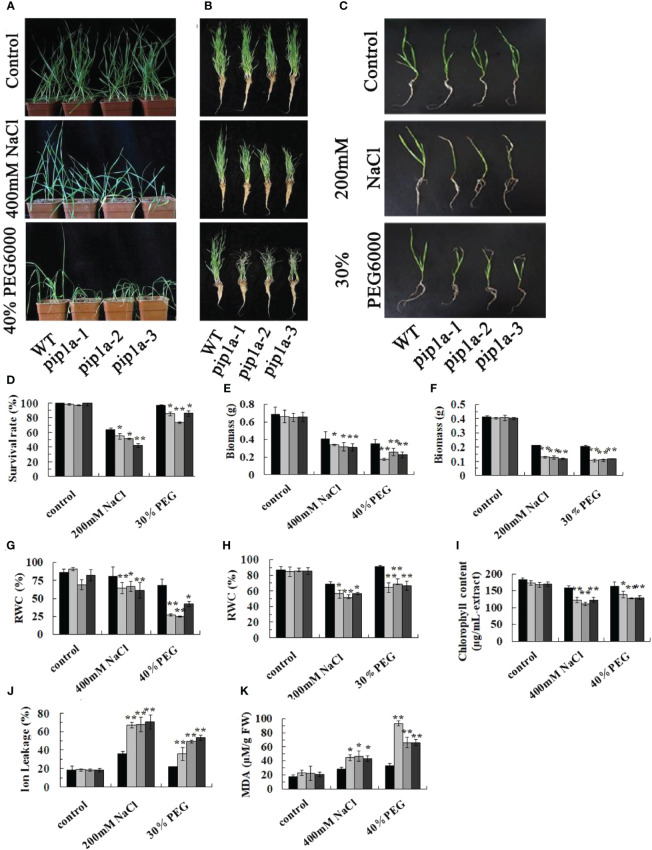
Phenotypic Responses of PIP1A Knockout to High Salt and Drought-Induced Stress. **(A)** Performance of WT, pip1a-1, -2, and -3 plants in potted soil under these stress conditions. Ten-day-old WT and mutant seedlings were exposed to 0 or 400 mM NaCl and 40% PEG for 15 days before being photographed. **(B)** Photographs of washed wheat seedlings after the treatments as described in **(A)**. **(C)** Performance of WT, pip1a-1, -2, and -3 plants grown using hydroponic culture under high salt and drought conditions. Ten-day-old WT and mutant seedlings were grown in half-strength Hoagland liquid medium with 0 or 200 mM NaCl and 30% PEG added, and cultured at 22°C. Photographs were taken after 8 days of seedling growth. **(D)** Survival rates of WT and pip1a mutants under normal and induced stress conditions in hydroponic culture. Biomass under soil and hydroponic conditions are shown in **(E, F)** respectively. Relative water content (RWC) under soil and hydroponic culture conditions are shown in **(G, H)** respectively. Chlorophyll content **(I)** and MDA content **(K)** of WT and pip1a plants in potted soil under normal and induced stress conditions. Ion leakage (IL) of WT and pip1a mutants under normal and induced stress conditions in hydroponic culture **(J)**. Data are presented as the mean ± standard deviation of three replicates. Asterisks indicate significant differences between WT and transgenic lines (*P < 0.05, **P < 0.01).

To investigate the function of *TaPIP1A*, a mutagenized library of KN9204 (WT) mutants generated by ion beam irradiation was utilized for screening pip1a mutants. Three pip1a knockout lines, designated as pip1a-1, pip1a-2, and pip1a-3, were successfully identified based on PCR deletion products. These pip1a mutants were subjected to two rounds of backcrossing with KN9204, and their progeny were utilized for genetic studies. There were no significant differences observed in flowering time or morphology between KN9204 and the mutant lines. All four lines showed robust growth in half-strength Hoagland liquid medium or soil, with indistinguishable phenotypes in their seedling stage. However, under NaCl or PEG-induced stress, mutant seedlings were more prone to wilting compared to WT (**Figures 4A–C**). Additionally, the survival rate (**Figure 4D**), biomass (**Figures 4E, F**), and relative water content [RWC] (**Figures 4G, H**) of pip1a were lower than the control group. The leaf tips of the mutant lines yellowed faster compared to WT plants (**Figure 4C**), and the chlorophyll content in the mutant lines was lower than in WT (**Figure 4I**). Ion leakage (IL), as a key marker of cell membrane damage, was significantly higher in the mutant lines than in WT, indicating more severe cell membrane damage in the mutant plants under abiotic stress compared to WT plants (**Figure 4J**). Furthermore, the malondialdehyde (MDA) content in pip1a seedlings was approximately twice that of WT (**Figure 4K**). These results suggest that *TaPIP1A* plays a positive role as a factor in wheat’s adaptive response to high salt and drought-induced stress.

### Overexpression of *TaPIP1A* in Arabidopsis enhances tolerance to high salt and drought stress

**Figure 5:**
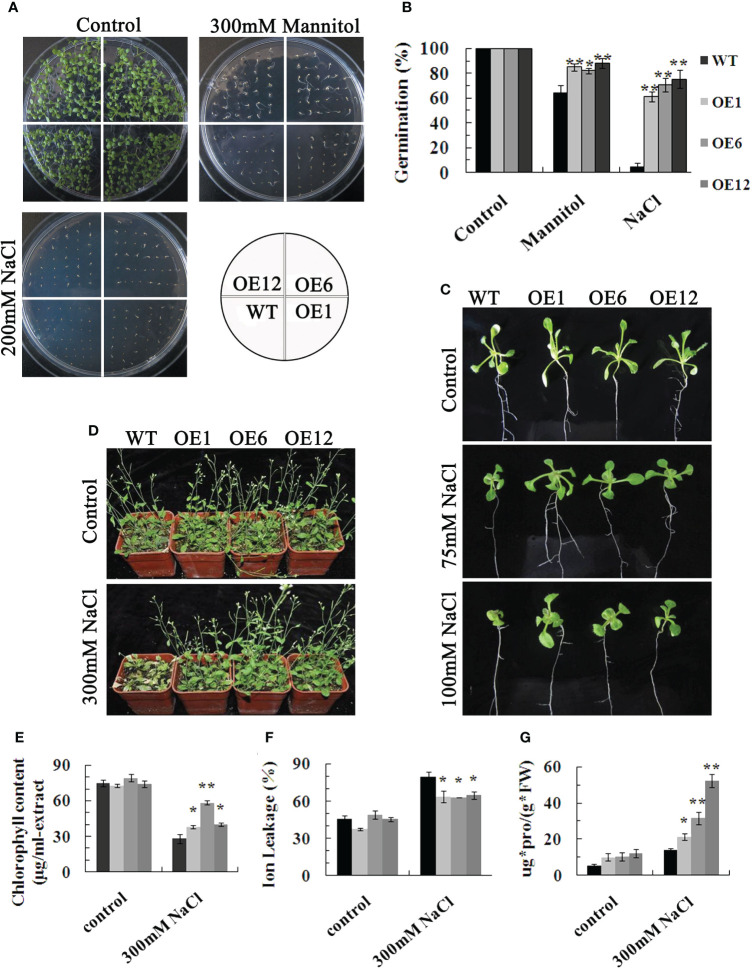
Phenotypic Characterization of Arabidopsis Plants Overexpressing *TaPIP1A*. **(A)** Phenotypic comparison of WT and transgenic plants during seed germination stage under normal, high salt, and drought conditions. Seeds of WT and transgenic lines were sowed on MS medium containing 0 or 200 mM NaCl and 300 mM mannitol, respectively, and cultured for nine days; **(B)** Germination rates of WT and transgenic seeds under normal, high salt, and drought conditions; **(C)** Phenotypic analysis of WT and transgenic plants at the seedling stage under normal and high salt conditions. Three-day-old transgenic seedlings were transferred to solid MS medium with 0, 75, or 100 mM NaCl and grown for eight days; **(D)** Phenotypic characterization of WT and transgenic mature plants grown under normal and high salt conditions. Four-week-old transgenic plants were exposed to 0 or 300 mM NaCl for eight days before photographing, followed by leaf harvest for measuring chlorophyll content **(E)**, IL **(F)**, and proline content **(G)**.WT: Wild type; OE1, OE6, and OE12: Transgenic lines overexpressing *TaPIP1A*. Data are presented as means ± standard deviation of three independent replicates. Asterisks denote significant differences between WT and transgenic lines (*P < 0.05, **P < 0.01).

To further investigate the function of *TaPIP1A*, transgenic Arabidopsis plants were generated, and three independent overexpression lines, OE-1, -6, and -12, were selected. There was no significant difference in seed germination rates between WT and OE series on half-strength MS medium; however, when exposed to 300 mM mannitol or 200 mM NaCl in the medium, seeds of the OE series exhibited a higher germination rate compared to WT (**Figures 5A, B**). Regardless of growth on MS agar plates or in soil without stress treatment, there were no obvious phenotypic differences between the OE series and WT plants (**Figures 5C, D**). However, when subjected to 75 or 100 mM NaCl, the leaves of OE series plants were larger than those of WT plants (**Figure 5C**). Additionally, the growth differences between OE and WT plants were more pronounced at higher NaCl concentrations. After four weeks of high salt stress induction, WT plants showed severe yellowing and wilting in their rosette leaves, while the leaves of OE plants remained green (**Figure 5D**). Moreover, the chlorophyll content in the OE group was significantly higher compared to the wild-type group (**Figure 5E**). Under high salt-induced stress, the OE line exhibited significantly lower ion leakage (IL) values than the WT plants (**Figure 5F**), and the proline content in the leaves of the OE line was higher than that in the leaves of the WT plants (**Figure 5G**). These results demonstrate that the overexpression in Arabidopsis significantly enhances tolerance to these abiotic stresses, highlighting the crucial positive regulatory role of *TaPIP1A* in stress response.

### The involvement of *TaPIP1A* in regulating the expression of stress-responsive genes in Arabidopsis

**Figure 6:**
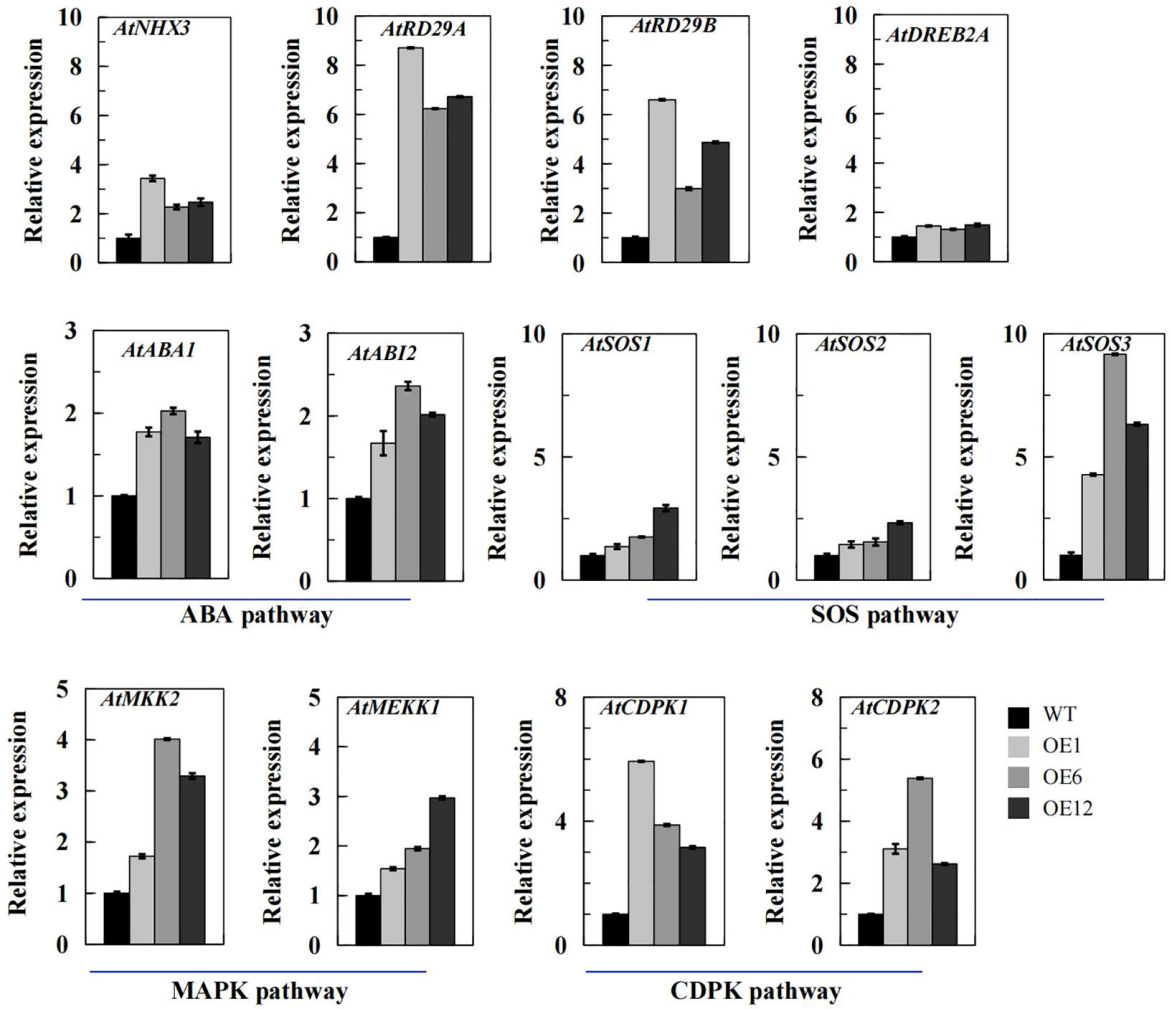
qRT-PCR Analysis of Stress-Related Gene Transcription Levels in Arabidopsis Plants. *Arabidopsis thaliana* seedlings were grown on MS medium for one week, then transferred to pots filled with vermiculite and soil (2:1 ratio) and maintained at 22°C. At four weeks old, the plants were subjected to 300 mM NaCl for eight days to induce stress. The transcription levels of stress-responsive genes were determined using qRT-PCR with specific primers, using Arabidopsis Actin gene (*At3g18780*) as an internal control for expression. The transcription levels were quantified using the 2^–ΔΔCT^ method for comparative analysis. Vertical bar graphs depict the relative transcription levels, with error bars representing standard deviation (SD). Each experiment was conducted in triplicate.

By analyzing the expression patterns of 13 typical abiotic stress-responsive genes in transgenic Arabidopsis plants overexpressing *TaPIP1A*, including four stress-responsive genes: *AtNHX3*, *AtRD29A*, *AtRD29B*, and *AtDREB2A* (**Figures 6A–D**); two ABA synthesis or response-related genes: *AtABA1* and *AtABI2* (**Figures 6E, F**); three SOS signal transduction-related genes: *AtSOS1*, *AtSOS2*, and *AtSOS3* (**Figures 6G–I**); two MAPK signal pathway-related genes: *AtMKK1* and *AtMEKK2* (**Figures 6J, K**); and two CDPK signal pathway-related genes: *AtCDPK1* and *AtCDPK2* (**Figures 6L, M**). The results of qRT-PCR indicate that *TaPIP1A* may modulate plant stress responses by affecting multiple signaling pathways, as observed in transgenic Arabidopsis plants overexpressing *TaPIP1A*. The findings suggest that the overexpression of *TaPIP1A* significantly impacts the expression of multiple stress-responsive genes in Arabidopsis, involving ABA synthesis, SOS signal transduction, MAPK and CDPK signaling pathways, revealing the crucial role of *TaPIP1A* in coordinating multiple signaling pathways during the response to abiotic stress.

### Interaction between *TaPIP1A* and TaPIP2-3 in regulating wheat response to stress

Using *Arabidopsis thaliana* AtPIP1A (At3g61430), AtPIP1C (At1g01620), AtPIP1;4 (At4g00430), and AtPIP1;5 (At4g23400) as probes, proteins interacting with *TaPIP1A* were identified in the String database (http://string-db.org/). Results indicated that RD28 (At2g37180) is a probable interacting partner of *TaPIP1A* (**Supplementary Figure 3**). RD28, a member of the PIP2 family, is known to be involved in dehydration response (Javot et al., 2003). To confirm this interaction both *in vitro* and *in vivo*, homologous genes encoding RD28 in wheat were identified through the EMBL-EBI database (http://plants.ensembl.org/index.html). TaPIP2-3 (KU366818) was cloned for subsequent analysis.

**Figure 7:**
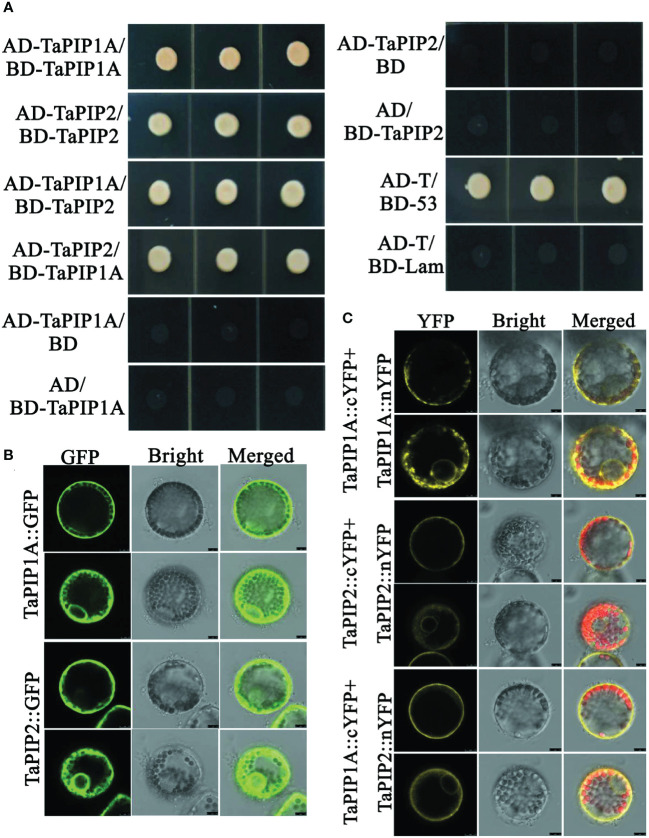
Interaction Analysis between *TaPIP1A* and TaPIP2-3 in Wheat Leaf Protoplasts. **(A)** Yeast two-hybrid experiments. Yeast cells transformed with plasmid combinations were cultured on QDO plates (SD/–Ade/–His/–Leu/–Trp) for three days. Co-transformed pGADT7-T and pGBKT7-53 constructs were used as positive controls, while co-transformed pGADT7-T and pGBKT7-Lam constructs were used as negative controls. Each experiment was repeated three times, and all replicates consistently yielded similar results; **(B)** Localization of TaPIP1A and TaPIP2-3 in wheat leaf mesophyll protoplasts. Protoplasts were transformed with gene fusion constructs, and after 12 hours, cells were observed using confocal microscopy, with GFP fluorescence excited by 488 nm light. **(C)** BiFC experiments in wheat leaf mesophyll protoplasts. Protoplasts were transformed with recombinant plasmids, and after 12 hours, cells were observed using laser scanning confocal microscopy. Chlorophyll autofluorescence was excited by 543 nm light, while YFP fluorescence was excited by 488 nm light. Protoplasts transiently co-expressing fusion proteins displayed as confocal (left panel), bright-field (middle panel), and merged (right panel) images.

Yeast two-hybrid system was employed to detect the interaction between *TaPIP1A* and TaPIP2-3. Full-length cDNAs of *TaPIP1A* and TaPIP2-3 were cloned into pGADT7 and pGBKT7 vectors, respectively. Pairing combinations of AD-*TaPIP1A* with BD-TaPIP2-3 and AD-TaPIP2-3 with BD-*TaPIP1A* showed clear positive interaction on QDO media (SD/-Leu/-Trp/-His/-Ade). Using WT yeast cells as a negative control, cells transfected with plasmids encoding AD-*TaPIP1A*, AD-TaPIP2-3, BD-*TaPIP1A*, and BD-TaPIP2-3 fusion proteins did not exhibit any DNA binding or self-activation characteristics (**Figure 7A**).

Further validation of the interaction between *TaPIP1A* and TaPIP2-3 was performed using BiFC (bimolecular fluorescence complementation) analysis with protoplasts isolated from wheat leaves. Yellow fluorescence indicated that *TaPIP1A* interacts with TaPIP2-3 at the plasma membrane (PM) and nuclear membrane (NM), consistent with their subcellular localization. Additionally, interactions of *TaPIP1A*-*TaPIP1A* and TaPIP2-3-TaPIP2-3 were observed in PM and NM (**Figures 7B, C**; **Supplementary Figure 4**). Experimental findings revealed a direct interaction between *TaPIP1A* and TaPIP2-3, supported by their localization in the plasma and nuclear membranes, uncovering the mechanism by which *TaPIP1A* functions in stress response by interacting with members of the PIP2 family in wheat.

### *TaWRKY71* enhances *TaPIP1A* expression by directly binding to its promoter

**Figure 8:**
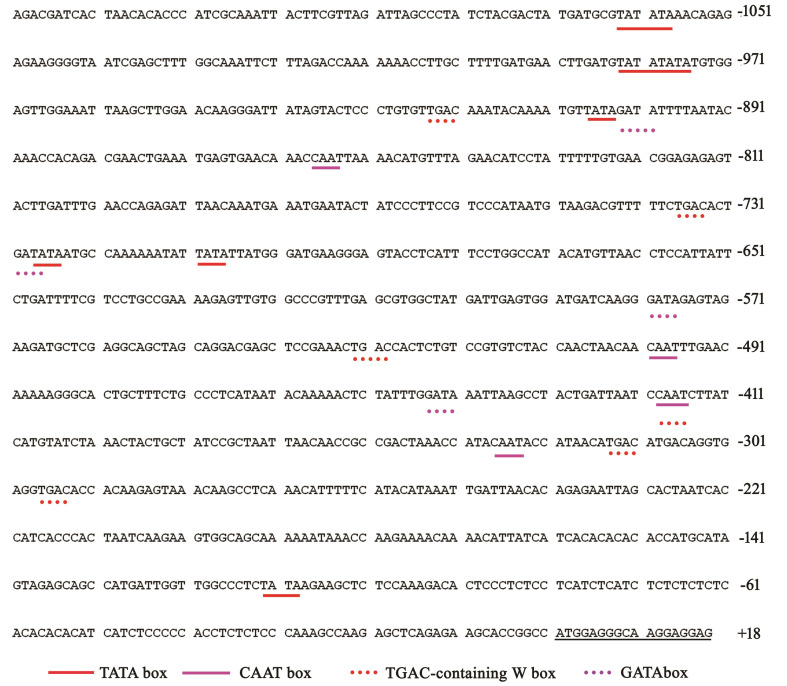
Identification of Cis-Elements in the *TaPIP1A* Promoter Through Scanning with Specific Probes in the PlantCARE and NEW PLACE Databases. *TaPIP1A* encompasses a 1.5 kb 5’-flanking region containing its promoter. The translation initiation site is denoted as +1. Partial TATA and CAAT boxes, W boxes containing TGAC, and GATA boxes are highlighted with distinct colored underlines (color figure available online).

To identify the transcription factor (TF) regulating *TaPIP1A* expression, two bioinformatics tools, New PLACE and PlantCARE, were used to analyze the promoter of *TaPIP1A* [KU366811], revealing seven WRKY71 binding cis-elements “TGAC” (**Figure 8**; **Supplementary Table 3**). The transcription factor *TaWRKY71*, known for its involvement in response to abiotic stress, and its potential role in the transcriptional regulation of TaPIP1, was identified (**Supplementary Figure 5**). Subsequently, *TaWRKY71* (KU366821) was cloned from KN9204. The SMART software (http://smart.embl-heidelberg.de/) identified the conserved WRKY domain within the TaWRKY71 protein (**Supplementary Figure 6**).

**Figure 9:**
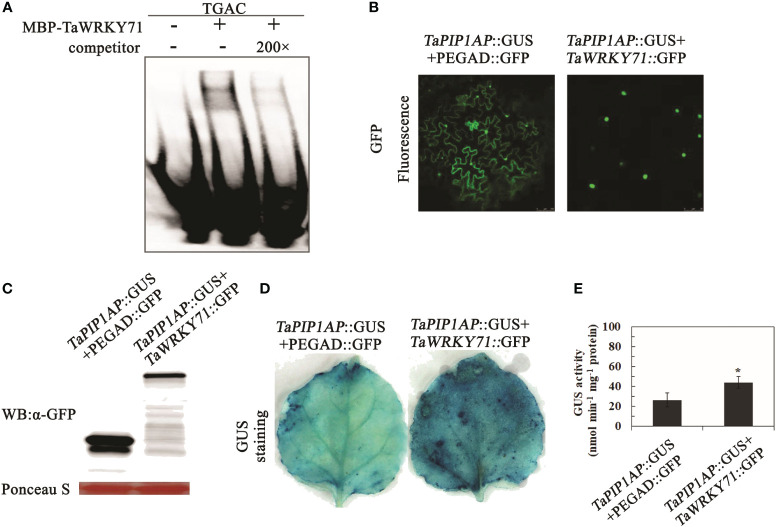
Interaction of *TaWRKY71* with the Promoter of *TaPIP1A* Leading to Its Upregulation. **(A)** EMSA was conducted to confirm binding in the presence (+) or absence (-) of 200-fold unlabeled competitor, protein incubated with labeled probe. The WRKY71 binding DNA sequence served as a negative control. The underlined sequence (TGAC) indicates cis-elements within the promoter region bound by WRKY71; **(B)** Subcellular localization of *TaWRKY71* and TaMYB1 in tobacco (*N. benthamiana*) leaf mesophyll cells. These cells were transformed with the dual-gene fusion constructs and observed under confocal microscopy 48 hours post-transformation. Images displayed are confocal, bright-field, and merged. GFP fluorescence was excited with 488 nm light; **(C)** Western blot analysis of fused proteins in leaf mesophyll cells. Loading amount for each sample was adjusted based on relative protein concentration determined by Ponceau staining (in red); **(D, E)** Upregulation of *TaPIP1A* promoter activity by *TaWRKY71* in tobacco leaves. Leaf mesophyll cells were transformed with dual-gene fusion constructs. GUS staining was performed 48 hours post-transformation **(D)**, and GUS activity was measured **(E)**. Data represent the mean ± standard deviation of three replicates. Asterisks indicate significant difference between WT and transgenic lines (*P < 0.05, **P < 0.01).

For the EMSA experiment, a probe containing four core TGAC elements from *TaWRKY71* as the P-W probe was designed (**Supplementary Table 1**). *TaWRKY71* was able to bind to P-W, but upon addition of a 200-fold concentration of unlabeled competitor, the signal of the binding complex weakened (**Figure 9A**). These findings indicate *TaWRKY71*’s capability to bind to specific cis-elements within the *TaPIP1A* promoter.

To determine the impact of *TaWRKY71* on *TaPIP1A* expression, agrobacterium-mediated transformation was used to introduce two constructs into tobacco (*N. benthamiana*) leaves. The constructs included p*TaPIP1A*::GUS with pEGAD-GFP, as well as p*TaPIP1A*::GUS with pEGAD-*TaWRKY71*::GFP. Confocal microscopy revealed the specific nuclear localization of *TaWRKY71* in leaf cells (**Figure 9B**), consistent with the typical characteristics of a transcription factor. Furthermore, Western blot analysis confirmed the expression of GFP and *TaWRKY71*::GFP in tobacco leaves (**Figure 9C**). In GUS histochemical staining, leaves expressing p*TaPIP1A*::GUS/pEGAD-GFP exhibited a weak GUS signal, whereas those expressing p*TaPIP1A*::GUS/*TaWRKY71*::GFP displayed a relatively intense staining (**Figure 9D**). Five series with high GUS expression from each construct were chosen for quantitative analysis based on histochemical analysis. In line with the GUS staining results, GUS expression was significantly activated by *TaWRKY71*, showing approximately a 1.7-fold increase in GUS activity in transgenic tobacco leaves (**Figure 9E**). The results indicate that the transcription factor *TaWRKY71* can directly bind to the specific cis-element “TGAC” within the *TaPIP1A* promoter, thereby activating *TaPIP1A* expression and elucidating the signal transduction mechanism of *TaWRKY71*-*TaPIP1A* in wheat stress tolerance.

## Discussion

Wheat, as one of the most crucial cereal crops globally, plays a direct role in global food security (Kumar et al., 2023; Zhang et al., 2024). However, abiotic stresses, particularly high salinity and drought conditions, significantly constrain wheat growth and yield (Schiavi et al., 2021). In this context, gaining a deep understanding of how wheat regulates and adapts to these stress conditions is of paramount importance for enhancing stress tolerance and ensuring food security (Du et al., 2023; Zuluaga et al., 2023). In recent years, an increasing number of studies have focused on the functionality of aquaporin proteins in plants, specifically the role of PIP family members in regulating plant water balance and enhancing stress tolerance (Guo et al., 2022; Song et al., 2022; Groszmann et al., 2023). This study centers on the role of the *TaPIP1A* gene in wheat and its expression regulation under high salinity and drought stress, revealing its potential mechanisms in enhancing wheat stress tolerance and offering a scientific foundation for further molecular breeding.

*TaPIP1A*, as a critical member of the PIP family, plays a vital role in maintaining plant cell membrane water balance (Jiang et al., 2022; Li et al., 2022b; Choong et al., 2023). Through the cloning and functional analysis of *TaPIP1A* and its homologous genes *TaPIP1B* and *TaPIP1D*, this study uncovers the unique function of *TaPIP1A* in wheat, particularly in playing a key role against high salinity and drought stress. Previous research has mainly focused on the functions of PIP family proteins under general physiological conditions, with limited studies on their roles under specific abiotic stress conditions (Jiang et al., 2022; Li et al., 2022b; Choong et al., 2023). Our findings demonstrate that *TaPIP1A* can interact with TaPIP2-3, localizing to the plasma and nuclear membranes, providing a new perspective on understanding the molecular mechanisms of PIP proteins in plants responding to abiotic stresses.

The role of transcription factor *TaWRKY71* is revealed for the first time in this study to directly regulate the expression of *TaPIP1A* by specifically binding to cis-elements in the *TaPIP1A* promoter, thereby impacting wheat’s tolerance to high salinity and drought stress. This discovery not only expands our understanding of the involvement of WRKY transcription factors in plant stress responses but also unveils a specific molecular mechanism by enhancing plant stress tolerance through regulating aquaporin gene expression. In previous studies, WRKY family members were mainly considered to respond to abiotic stresses by participating in plant defense responses and growth regulation, with relatively fewer studies on their direct regulation of specific target genes (Abdullah-Zawawi et al., 2021; Wani et al., 2021; Wang et al., 2023a).

In comparison with previous studies, this research not only elucidates the specific function and mechanism of *TaPIP1A* in wheat but also demonstrates the regulatory role of *TaWRKY71* as a transcription factor in this process. Through studies involving *TaPIP1A* gene knockout and overexpression, we further confirm its crucial role in enhancing wheat’s tolerance to high salt and drought stress (Song et al., 2020). Additionally, this study reports for the first time the interaction between *TaPIP1A* and TaPIP2-3, providing significant clues for understanding the complex network of aquaporins in regulating plant stress responses. In contrast to existing literature, this study not only offers new insights into the function of *TaPIP1A* but also reveals the role of *TaWRKY71* in regulating its expression, providing valuable information for a deeper exploration of the molecular mechanisms behind plant responses to abiotic stress.

Methodologically, this study employed a range of advanced molecular biology and biochemistry techniques, such as gene knockout, overexpression, electrophoretic mobility shift assay (EMSA), and β-glucuronidase (GUS) reporter gene analysis, ensuring the accuracy and reliability of the research results. Particularly noteworthy is the validation through EMSA experiments of the direct interaction between *TaWRKY71* and the *TaPIP1A* promoter, offering compelling evidence for understanding how transcription factors precisely regulate target genes. By revealing the roles of *TaPIP1A* and *TaWRKY71* in enhancing wheat’s tolerance to high salt and drought stress, this study presents novel molecular targets for improving wheat’s stress resilience. This achievement not only advances our understanding of the mechanisms by which plants respond to abiotic stress but also holds significant practical value. Building upon these findings, novel wheat varieties can be developed through molecular breeding techniques to better adapt to adverse environmental conditions, thereby enhancing wheat productivity and stability, with positive implications for global food security.

In summary, this study unveils, for the first time, how *TaPIP1A* promotes wheat’s tolerance to drought and high salt-induced stress through interaction with TaPIP2-3, under the regulation of the transcription factor *TaWRKY71*. These discoveries offer potential genetic pathways for enhancing stress tolerance in wheat and other crops. Nevertheless, the study acknowledges limitations, including the lack of broader stress condition tests and on-field validation. Future research should delve into the roles of *TaPIP1A* and *TaWRKY71* under different abiotic stress conditions and explore their interactions with other potential regulators in wheat stress responses. Furthermore, validating the practical application of these molecular mechanisms through more field experiments will be a crucial direction for future research. A deeper understanding of these molecular regulatory networks not only sheds light on the intricate mechanisms by which plants adapt to environmental stress but also provides a scientific basis for formulating effective crop improvement strategies.

While this study offers new insights into the roles of *TaPIP1A* and *TaWRKY71* in wheat stress response, many questions remain unanswered. For instance, do *TaPIP1A* and *TaWRKY71* also regulate other stress response pathways in wheat? How do their expression patterns in different growth stages and tissues of wheat affect stress responses? Answers to these questions will further enrich our understanding of plant stress response mechanisms. Additionally, future studies should consider utilizing gene editing technologies such as CRISPR/Cas9 for precise control of *TaPIP1A* and *TaWRKY71* expression to confirm their specific roles in enhancing wheat stress tolerance. This approach can further explore the potential application value of these genes and pave the way for developing wheat varieties more resilient to environmental stress.

## Conclusion

**Figure 10:**
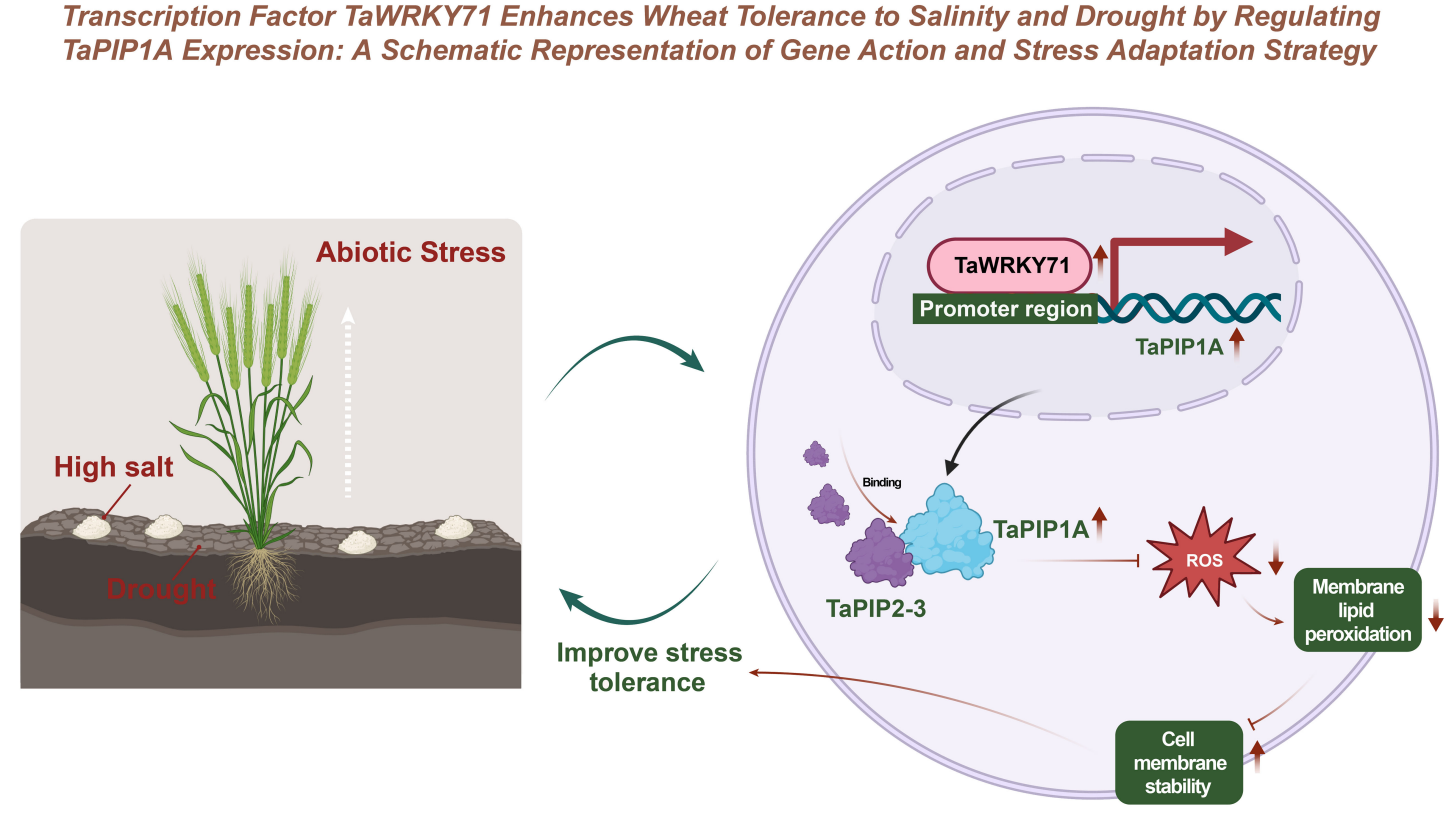
Enhancing Salt-Alkali and Drought Tolerance in Wheat through Transcription Factor *TaWRKY71* Regulation of *TaPIP1A* Expression: Gene Function and Stress Response Strategy Schematic Diagram.

This study reveals the underlying mechanisms by which the intrinsic *TaPIP1A* protein in wheat plays a positive role in plant responses to abiotic stress, particularly high salt and drought stress (**Figure 10**). *TaWRKY71* upregulates the expression of *TaPIP1A* by directly binding to its promoter, and *TaPIP1A* enhances wheat tolerance to high salt and drought stress through its interaction with TaPIP2-3. Furthermore, the overexpression of *TaPIP1A* in Arabidopsis further confirms its significant role in maintaining cell membrane integrity and enhancing stress tolerance.

Additionally, this study demonstrates the involvement of *TaPIP1A* in regulating the expression of stress-responsive genes and identifies specific cis-elements in its promoter using bioinformatics tools, providing new insights into the roles of *TaPIP1A* and its homologous genes in plant adaptive responses. The interaction between *TaPIP1A* and TaPIP2-3, along with their localization in the plasma membrane and nuclear membrane, elucidates their critical roles in regulating plant water channel activity and improving plant responses to stress.

In conclusion, this study not only elucidates the mechanism by which *TaPIP1A* regulates plant responses to high salt and drought stress in wheat but also offers new genetic resources for improving stress tolerance in wheat and other crops through molecular breeding techniques.

## Data Availability

The original contributions presented in the study are included in the article/**Supplementary Material**. Further inquiries can be directed to the corresponding authors.

## Supplementary material

Supplementary Figure 1 Chromosome distribution and tissue-specific expression of the wheat Pip1 gene family. **(A)** Chromosomal localization of the Pip1 gene family in the wheat genome. **(B)** Heatmap showing the expression patterns of the Pip1 gene family in different wheat tissues (roots, leaves, stems). The color gradient in the heatmap represents the level of expression, with dark colors indicating high expression and light colors indicating low expression.

Supplementary Figure 2 Supplementary Subcellular Localization of *TaPIP1A* Protein in Tobacco Leaf Mesophyll Cells and Transgenic Arabidopsis Roots via Immunofluorescence Analysis. The 35S::GFP and 35S::GFP::*TaPIP1A* fusion proteins were transiently expressed in tobacco leaf mesophyll cells and stably transformed in Arabidopsis. **(A)** GFP fluorescence in tobacco leaf mesophyll cells; **(B)** GFP fluorescence in transgenic Arabidopsis roots. Images show confocal, bright-field, merged, and PI-stained images. The GFP fluorescence was excited at a wavelength of 488 nm.

Supplementary Figure 3 Supplementary Protein-Protein Interaction Network of PIP1 Subunits Obtained Through STRING Interaction Analysis. Using homologous proteins from *Arabidopsis thaliana* as baits, the online software STRING was utilized to identify proteins interacting with *TaPIP1A*. **(A)** AtPIP1A (At3g61430), **(B)** AtPIP1C (At1g01620), **(C)** AtPIP1;4 (At4g00430), and **(D)** AtPIP1;5 (At4g23400) are depicted in a confidence view to display their interaction networks, with stronger associations represented by thicker lines.

Supplementary Figure 4 Supplementary Negative Control of BiFC Experiment in Wheat Leaf Protoplasts. Recombinant plasmids were co-transfected into wheat leaf protoplasts for 12 hours, followed by observation of the cells using laser scanning confocal microscopy. YFP fluorescence was excited at 488 nm, while chlorophyll autofluorescence was excited at 543 nm. The confocal (left panel), bright-field (middle panel), and merged (right panel) images show transient co-expression of the specified fusion proteins in wheat leaf protoplast cells. The experiment was repeated three times, and consistent results were obtained in all experiments.

Supplementary Figure 5 Supplementary Alignment of the TaPIP1 promoter from chromosome A, B, and D in hexaploid wheat. Sequences were aligned using the DNAMAN program. Nucleotide numbers are shown on the right. Light blue indicates the nucleotide substitutions are identical in all analyzed sequences.

Supplementary Figure 6 Supplementary Structural Analysis of *TaWRKY71* Using SMART Online Software.
