# Supplementary figures and images for: Overexpression of TaPIP1A enhances drought and salt stress tolerance in Arabidopsis: cross-species conservation and molecular dynamics
Source: Front Plant Sci. 2025 Jun 2;15:1425700. doi: 10.3389/fpls.2024.1425700 (PMC12172022; doi:10.3389/fpls.2024.1425700)

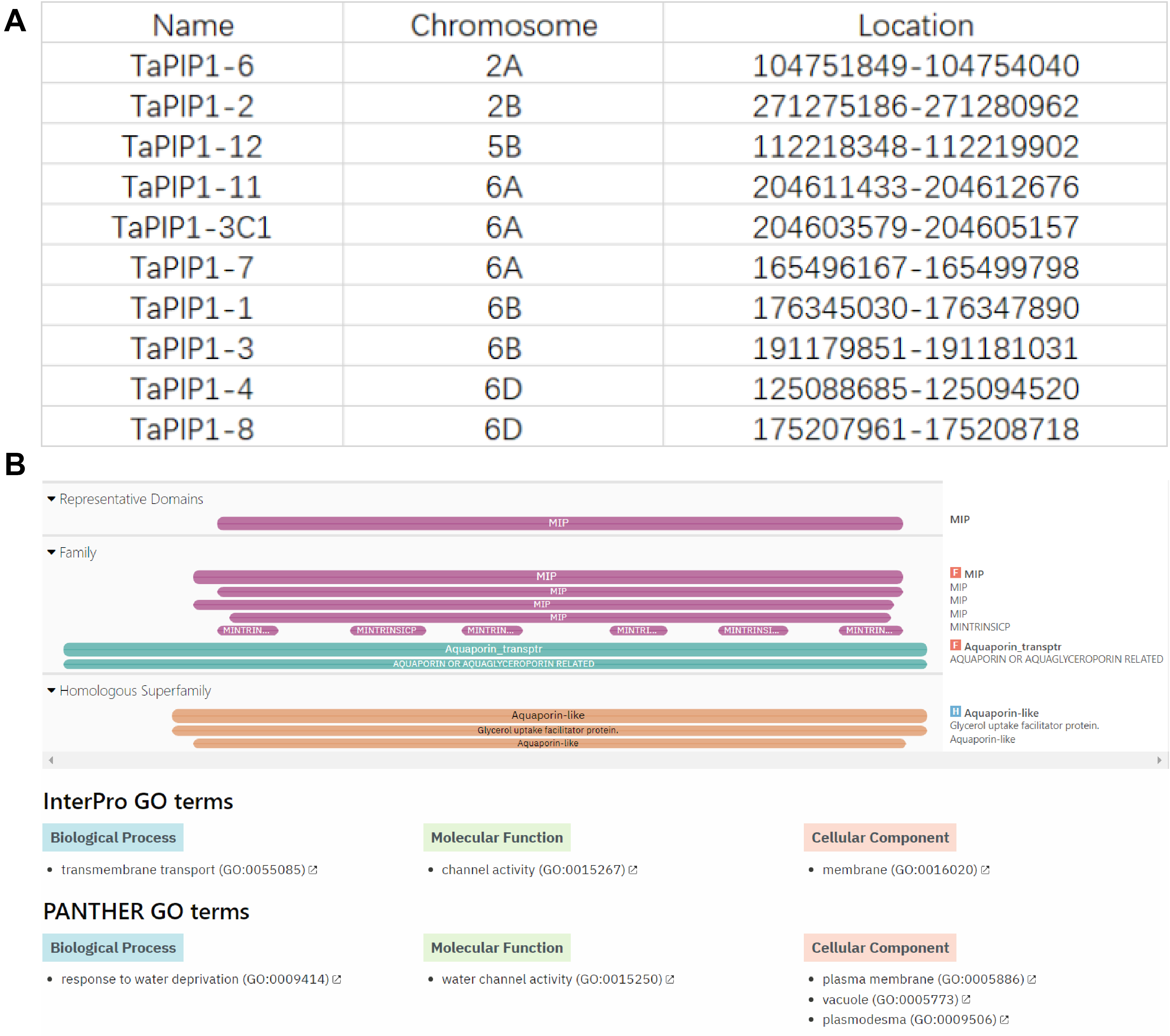

Supplement: Supplementary Figure 1 — Chromosome distribution and tissue-specific expression of the wheat Pip1 gene family. (A) Chromosomal localization of the Pip1 gene family in the wheat genome. (B) Heatmap showing the expression patterns of the Pip1 gene family in different wheat tissues (roots, leaves, stems). The color gradient in the heatmap represents the level of expression, with dark colors indicating high expression and light colors indicating low expression. [file Image1.jpeg]

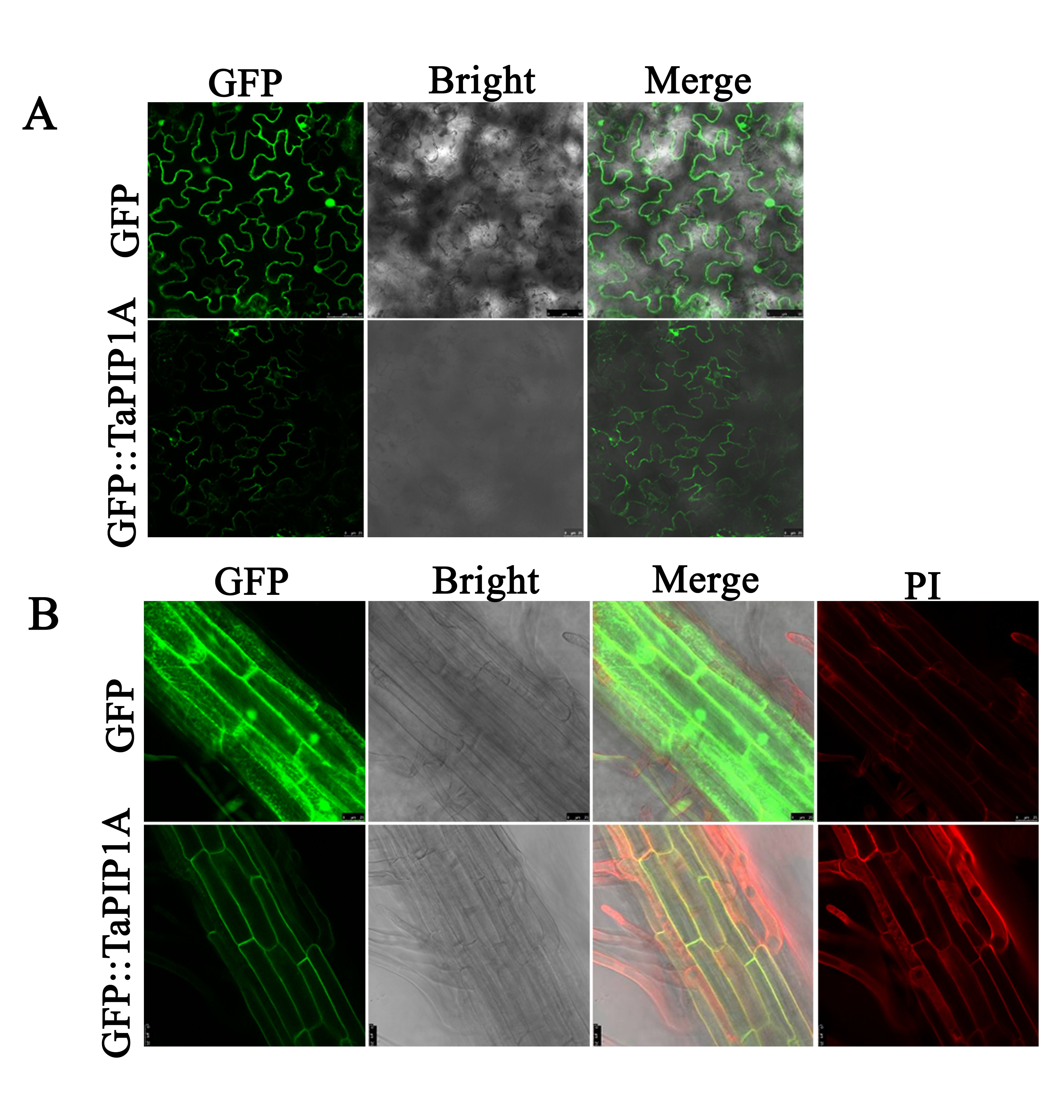

Supplement: Supplementary Figure 2 — Supplementary Subcellular Localization of TaPIP1A Protein in Tobacco Leaf Mesophyll Cells and Transgenic Arabidopsis Roots via Immunofluorescence Analysis. The 35S::GFP and 35S::GFP::TaPIP1A fusion proteins were transiently expressed in tobacco leaf mesophyll cells and stably transformed in Arabidopsis. (A) GFP fluorescence in tobacco leaf mesophyll cells; (B) GFP fluorescence in transgenic Arabidopsis roots. Images show confocal, bright-field, merged, and PI-stained images. The GFP fluorescence was excited at a wavelength of 488 nm. [file Image2.jpeg]

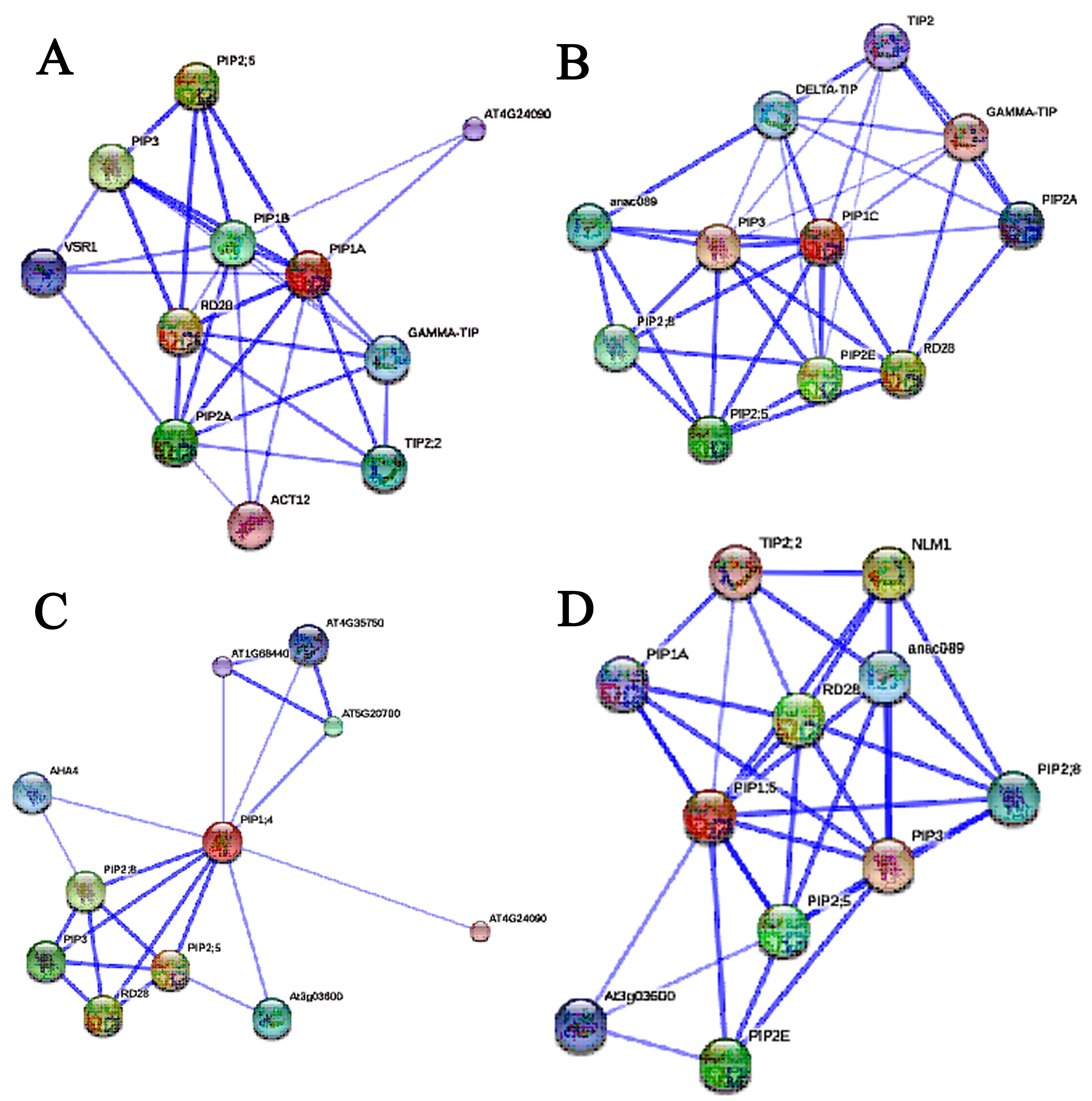

Supplement: Supplementary Figure 3 — Supplementary Protein-Protein Interaction Network of PIP1 Subunits Obtained Through STRING Interaction Analysis. Using homologous proteins from Arabidopsis thaliana as baits, the online software STRING was utilized to identify proteins interacting with TaPIP1A. (A) AtPIP1A (At3g61430), (B) AtPIP1C (At1g01620), (C) AtPIP1;4 (At4g00430), and (D) AtPIP1;5 (At4g23400) are depicted in a confidence view to display their interaction networks, with stronger associations represented by thicker lines. [file Image3.jpeg]

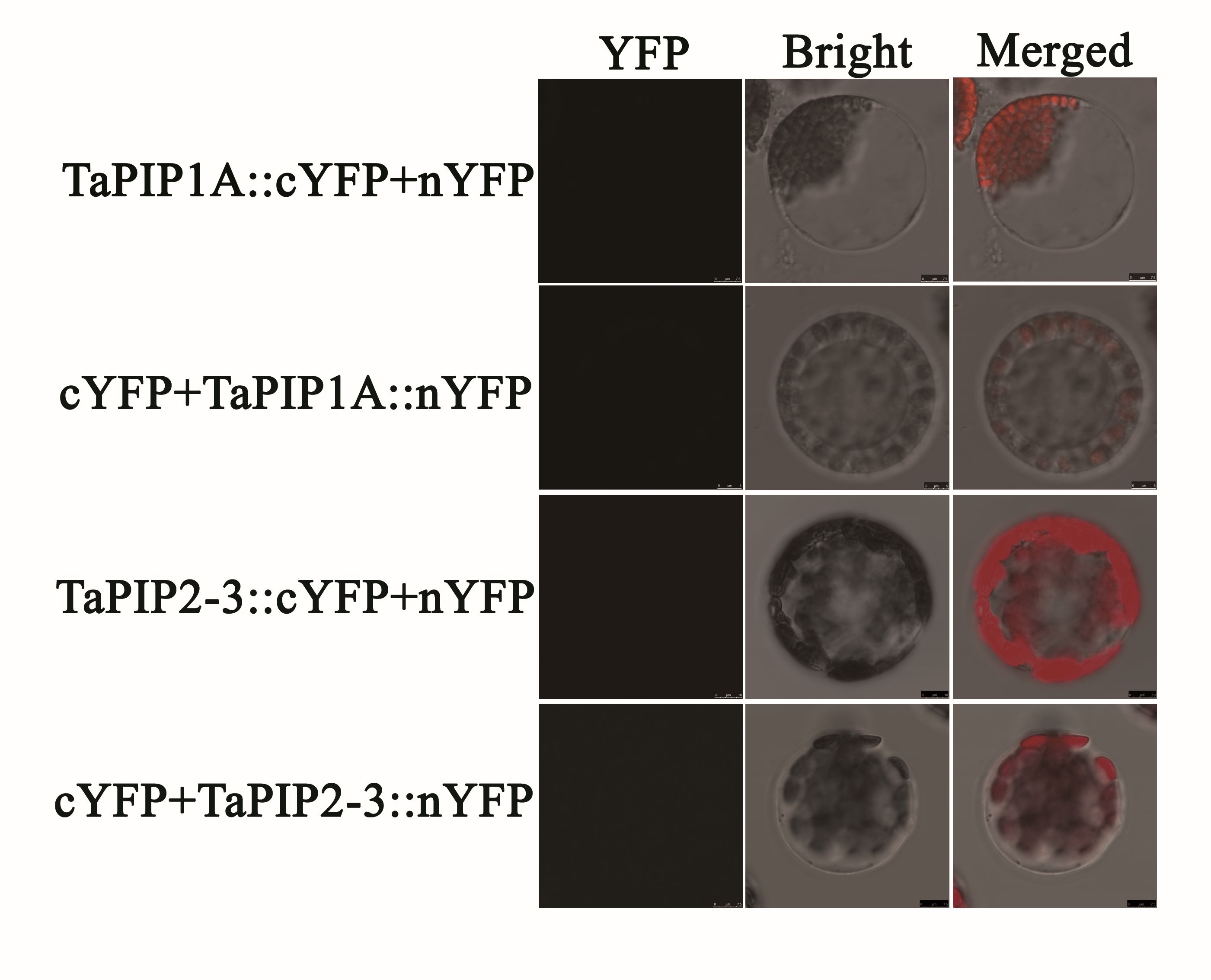

Supplement: Supplementary Figure 4 — Supplementary Negative Control of BiFC Experiment in Wheat Leaf Protoplasts. Recombinant plasmids were co-transfected into wheat leaf protoplasts for 12 hours, followed by observation of the cells using laser scanning confocal microscopy. YFP fluorescence was excited at 488 nm, while chlorophyll autofluorescence was excited at 543 nm. The confocal (left panel), bright-field (middle panel), and merged (right panel) images show transient co-expression of the specified fusion proteins in wheat leaf protoplast cells. The experiment was repeated three times, and consistent results were obtained in all experiments. [file Image4.jpeg]

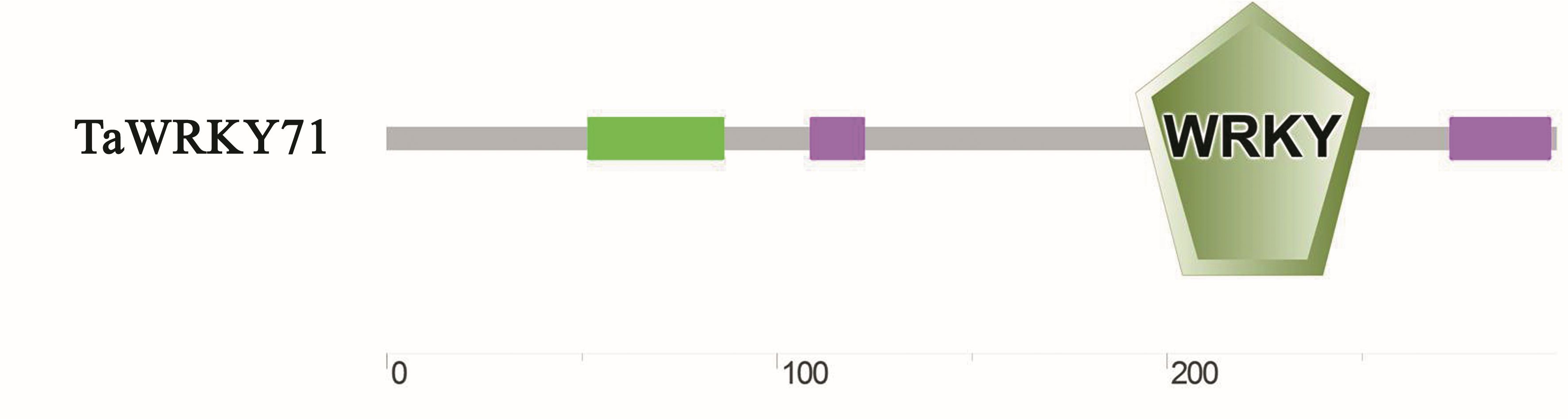

Supplement: Supplementary Figure 6 — Supplementary Structural Analysis of TaWRKY71 Using SMART Online Software. [file Image6.jpeg]
