# Supplementary material for: Overexpression of TaPIP1A enhances drought and salt stress tolerance in Arabidopsis: cross-species conservation and molecular dynamics
Source: Front Plant Sci. 2025 Jun 2;15:1425700. doi: 10.3389/fpls.2024.1425700 (PMC12172022; doi:10.3389/fpls.2024.1425700)
Supplement: Supplementary file 7 [file Table1.docx]

**Table S1. Primers used for gene cloning and functional analysis.**

| **Primer name** | ***Sequence (5→3’)*** |
| --- | --- |
| TaPIP1F | ATGGAGGGCAAGGAGGAGG |
| TaPIP1R | CTAGTCGCGGCTCTTGAAG |
| TaPIP2F | ATGGCCAAGGAGGTGAGCG |
| TaPIP2R | TCAGTTGCTCCGGCTGCTC |
| TaPIP1A-promoterF | TCTCTCCCAAAGCCAAGAGC |
| TaPIP1A-promoterR | CAGCGATAAGATTCCACCCG |
| TaPIP1B-promoterF  TaPIP1D-promoter | ATGGAGGGCAAGGAGGAGGA |
| TaPIP1B-promoterR  R | CTAGTCGCGGCTCTTGAAGG |
| TaPIP1D-promoterF | ATGGAGGGCAAGGAGGAGGA |
| TaPIP1D-promoterR | CTAGTCGCGGCTCTTGAAGGG |
| mPIP1AF | TCTCTCCCAAAGCCAAGAGC |
| mPIP1AR | CAGCGATAAGATTCCACCCG |
| EcoRI-PIP1AF | GAATTCATGGAGGGCAAGGAGGAGG |
| BamHI-PIP1AR | GGATCCCTAGTCGCGGCTCTTGAAG |
| BamHI-PIP1AF | GGATCCATGGAGGGCAAGGAGGAGG |
| StuI-PIP1AR | AGGCCTGTCGCGGCTCTTGAAGGG |
| BamHI-PIP2F | GGATCCATGGCCAAGGAGGTGAGCG |
| StuI-PIP2R | AGGCCTGTTGCTCCGGCTGCTCCG |
| BglII-WRKY71F | AGATCTATGGATCCATGGGTCAGCAG |
| SalI-WRKY71R | GTCGACGCCGCTGACGGCGGCC |
| XbaI-WRKY71R | TCTAGAGCCGCTGACGGCGGCC |
| EcoRI-PIP2F | GAATTCATGGCCAAGGAGGTGAGCG |
| BamHI-PIP2R | GGATCCTCAGTTGCTCCGGCTGCTC |
| PstI-PIP1ApromoterF | CTGCAGGCACACAAGTATTTATCGGTG |
| NcoI-PIP1ApromoterR | CCATGGGGCCGGTGCTTCTCTGAG |
| P-WF | CATGACATGACAGGTGAGGTGACACCACAAG |
| P-WR | CTTGTGGTGTCACCTCACCTGTCATGTCATG |
