# Supplementary material for: Overexpression of TaPIP1A enhances drought and salt stress tolerance in Arabidopsis: cross-species conservation and molecular dynamics
Source: Front Plant Sci. 2025 Jun 2;15:1425700. doi: 10.3389/fpls.2024.1425700 (PMC12172022; doi:10.3389/fpls.2024.1425700)
Supplement: Supplementary file 8 [file Table2.docx]

**Table S2. Primers used for qRT-PCR analysis.**

| **Primer name** | **Forward *（5’- 3’）*** | **Reverse *（5’- 3’）*** |
| --- | --- | --- |
| qTaActin | TGCTATCCTTCGTTTGGACCTT | AGCGGTTGTTGTGAGGGAGT |
| qPIP1A | CGGGCTAGGGGCTGAGATCG | CGCTTGGCGTCGGTGGC |
| qAtActin | TCGCTGACCGTATGAGCAAAG | TGTGAACGATTCCTGGACCTG |
| qAtNHX3 | TACCGCCATTTTAGTAGGAGCA | TAACTTGGAACCCAGCATTGA |
| qAtDREB2A | GTGACCTAAATGGCGACGAT | GCGGATCAAAACCACTTTGT |
| qAtRD29A | ATCACTTGGCTCCACTGTTGTTC | ACAAAACACACATAAACATCCAAAGT |
| qAtRD29B | GAATCAAAAGCTGGGATGGA | TGCTCTGTGTAGGTGCTTGG |
| qAtABA1 | GCAATGGTATGCATTTCACG | CATGCTTCATCAAGCTCCAA |
| qAtABI2 | GATGGAAGATTCTGTCTCAACGATT | GTTTCTCCTTCACTATCTCCTCCG |
| qAtSOS1 | GGAGATGCTTGATGAGGGC | GGAAATTGACATGTGGTTTTAGAC |
| qAtSOS2 | GGCTTGAAGAAAGTGAGTCTCG | GCTACATAGTTCGGAGTTCCACA |
| qAtSOS3 | CATTCACGGTAGAAGAAGTGGA | GCTTGGATGGAAGACACCTAA |
| qAtFAD5 | CAGAGACCCTCATAGCCCACTT | CATGCTTGTTTTCCCCATACAT |
| qAtSAD1 | GCGAACAATCCTTCACAG | CTTCGGGAGACCCACCT |
| qAtMKK2 | GATGAAGCAATTCGCAAGGCA | AAGATGGCAGAAAGATAGGAGTCAGG |
| qAtMEKK1 | GACGCATTTCGCTCCAAGTG | CAAGACGTTATGATAGCTCCTCCA |
| qAtCDPK1 | CGTTCGTCGTGAGGTAGCG | GTAATGTCCTCTAGCAACAATCCG |
| qAtCDPK2 | TACGAAGATGTATGGCGTGAGA | ATGACCTTTAGAAACAATCCGATC |
