# Supplementary material for: Overexpression of TaPIP1A enhances drought and salt stress tolerance in Arabidopsis: cross-species conservation and molecular dynamics
Source: Front Plant Sci. 2025 Jun 2;15:1425700. doi: 10.3389/fpls.2024.1425700 (PMC12172022; doi:10.3389/fpls.2024.1425700)
Supplement: Supplementary file 9 [file Table3.docx]

**Table S3. The predicted *cis*-elements of the promoter of *TaPIP1A*, *TaPIP1B* and *TaPIP1D*.**

| *Cis*-element | *TaPIP1A* | *TaPIP1B* | *TaPIP1C* | Function |
| --- | --- | --- | --- | --- |
| CAAT box | 6 | 13 | 8 | common *cis*-acting element in promoter and enhancer regions |
| TATA box | 21 | 13 | 25 | core promoter element around -30 of transcription start |
| MYC BOX | 7 | 8 | 3 | MYC binding site |
| TGAC-containing W box | 7 | 8 | 7 | WRKY71 binding site |
| MBSII | 5 | 4 | 4 | MYB1 binding site |
| G box | 2 | 1 | 1 | *cis*-acting regulatory element involved in light responsiveness |
| I- box | 1 | 1 | 1 | part of a light responsive element |
| GATA box | 4 | 3 | 4 | part of a light responsive element |
| GAG motif | 1 | 2 | 2 | part of a light responsive element |
| HSE | 1 | **0** | 0 | *cis*-acting element involved in heat stress responsiveness |
| GARE motif | 1 | 0 | 1 | gibberellins responsive element |
| ATCT motif | 1 | 0 | 1 | part of a conserved DNA module involved in light responsiveness |
| LTR | 2 | 0 | 1 | *cis*-acting element involved in low-temperature responsiveness |
| ABRE | 2 | 1 | 1 | *cis*-acting element involved in the abscisic acid responsiveness |
